# Patient-reported outcome measures after periodontal surgery

**DOI:** 10.1007/s00784-023-05362-y

**Published:** 2023-11-09

**Authors:** Ilham Mounssif, Valentina Bentivogli, Alexandra Rendón, Davide B. Gissi, Francesco Maiani, Claudio Mazzotti, Monica Mele, Matteo Sangiorgi, Giovanni Zucchelli, Martina Stefanini

**Affiliations:** Department of Biomedical and Neuromotor Sciences, University of Bologna, Bologna, Italy

**Keywords:** OHIP, OHIP-14, Questionnaire, PROMS, Periodontal surgery, VAS, Postoperative morbidity

## Abstract

**Objectives:**

The present study aimed to explore the impact of different periodontal surgical treatments on the quality of life and postoperative morbidity.

**Materials and methods:**

The present study is a single-center, prospective, observational cohort trial. One hundred fifty-five patients, referred to the Periodontal Department of Bologna University who needed periodontal surgical treatment, were recruited. The self-reported perception of the postoperative course was assessed using the following anonymous questionnaires: Italian oral health impact profile (I-OHIP-14), visual analog scale (VAS) to evaluate the intensity of the pain, and 5-point Likert scale.

**Results:**

Patients reported a mean OHIP-14 total score of 9.87±8.5 (range 0–42), significantly influenced by the female sex, flap extension, and periodontal dressing. A mean VAS score of 2.96±2.39 (range 0–9) was calculated, and was found to be influenced by the presence of vertical releasing incisions and palatal flap extension. Of the 155 subjects, 40 (25.8%) patients reported bleeding as a post-surgical complication, 96 (61.9%) swelling, 105 (67.7%) eating discomfort, and 44 (28.4%) reported speech discomfort.

**Conclusions:**

Within the limitations of the nature of the present study, periodontal surgical procedures have a low impact on patients’ quality of life evaluated through the OHIP-14 and VAS pain questionnaires.

**Clinical relevance:**

Periodontal surgical procedures are safe procedures, with a limited duration of postoperative discomfort as well as the incidence of complications.

**Supplementary Information:**

The online version contains supplementary material available at 10.1007/s00784-023-05362-y.

## Introduction

Due to the nature of periodontal procedures, even though they are common practice, there is usually a latent risk of developing postoperative morbidity—defined as a condition of being diseased [1]—in the form of infection, swelling, and pain, which represents a matter of concern for both the practitioner and the patient [2].

In general, the incidence rate of infection reported after periodontal surgery is very low, ranging between <1 and 4.4% without administration of antibiotics [3–6]. However, certain factors, such as duration of the surgical procedure [7–10], type of surgery (i.e., resective or mucogingival procedures) [7, 10–12], surgeon’s experience [13], and patient smoking habits [8], have been identified and correlated with a greater pain perception during the postoperative time. On this regard, the most commonly used tool to assess pain perception is the visual analog scale (VAS) which is considered valid, reproducible, and easy to be administered [14]. Its application, together with other assessment tools, has increased significantly along with the urgent need to include professionally derived patient-centered outcomes for the global evaluation of periodontal surgical procedures [15].

Patient-reported outcome measures (PROMs) are defined as any report of the status of a patient’s health condition that comes directly from the patient, without interpretation of the patient’s response by a clinician or others [16]. Focusing on this approach has been recognized as fundamental in order to assess the impact of the treatment regarding the individual set of concerns of the patient as well as its broader impact on health and well-being [15]. Although periodontal diseases and their treatments are not life-threatening, they can affect different domains of personal and interpersonal daily activities (ability to eat, speak, and socialize) with repercussions on the quality of life [17, 18]. In 1997, Slade et al. [19] introduced the Oral Health Impact Profile-14 (OHIP-14) (1997), a questionnaire-tool designed to provide a comprehensive disclosure of the dysfunction, discomfort, and disability attributed to oral conditions. The OHIP-14 measures the adverse impact of oral health conditions associated with teeth, mouth, or dentures on physical, psychological, and social dimensions [20]. This instrument has been thoroughly tested and recommended for its reliability and validity [21, 22], its responsiveness [23, 24], and its cross-cultural consistency [25].

Several studies have applied the OHIP-14 to assess the impact of non-surgical therapy on the quality of life [26–28], but to the best of our knowledge, only two papers [18, 29] have used said tool to evaluate the immediate postoperative effects of different surgical periodontal modalities (i.e., resective, regenerative, and mucogingival surgeries) on patient perception of quality of life.

Therefore, the present study aimed to explore the impact of different periodontal surgical treatments on the quality of life using a validated OHIP-14 for Italian people (IOHIP-14) [30] and to evaluate the prevalence and distribution of post-surgical complications.

## Materials and methods

### Study population

The present study was designed as a single-center, prospective, observational cohort trial to investigate the impact of periodontal surgical procedures on patient quality of life. This paper was written according to the STROBE statement for improving the quality of reports of observational trials. The study protocol was approved by the local ethics committee at Bologna, Italy (PG0027334/2017-16180). Patients referred to the Periodontal Department of Bologna University between April 2019 and December 2022 requiring periodontal surgical treatment were recruited; each patient contributed with one questionnaire. Patients (> 18 years) with uncontrolled systemic or local diseases or taking antibiotics in the past 6 months and those using any additional remedies other than the prescribed medication for post-surgical pain control were excluded.

All periodontal surgeries were performed by expert periodontists (>10 years of experience in periodontal surgery) and residents with 3 years of periodontal training and followed standard protocols under local anesthesia in an isolated surgical operatory setting. Standardized post-surgical instructions were given to all the participants, and they were advised to take painkillers when needed (Ibuprofen 600 mg tablet), while systemic antibiotics (amoxicillin+ clavulanic acid 875 mg+125 mg for 7 days) were prescribed according to the surgeon’s decision and patient’s medical history.

The patients were asked to refrain from using mechanical oral hygiene measures in the treated area for 2 weeks, during which they were instructed to rinse with 0.12% chlorhexidine digluconate for 1 min, twice daily. Sutures were removed 2 weeks after surgery in all cases.

### Investigator meeting and calibration

Prior to the start of the study, a calibration meeting was held with each examiner to standardize data acquisition and the assessment of study variables. Two examiners (IM, FM), who did not perform the surgeries, were designated to collect patient information in the records, to explain to patients how to fill out the morbidity questionnaires and collect them once returned, and to transfer the information unto the data extraction template (CRF). All collected data were stored in an electronic database.

### Post-surgical evaluation

The questionnaires were delivered by the investigators on the day of surgery, and patients were instructed to fill the questionnaire at day 7 after the surgery. The self-reported perception of the postoperative course was assessed using the following anonymous questionnaires:

- Oral health impact profile (OHIP-14, Italian Version) questionnaire was administered to investigate the impact of the received surgery on quality of life. The OHIP-14 scores can range from 0 to 56 and are calculated by adding the ordinal values for each of the 14 domains. Scores for individual domains can range from 0 to 5.
- Visual analog scale (VAS) was used to evaluate the intensity of the pain (0= no pain; 10=maximum pain);
- The presence or absence of bleeding, swelling, eating, and speech discomfort was recorded with dichotomous questions;
- 5-point Likert scale (far too little, too little, about right, too much, far too much) was used in case of a positive answer to the previously reported questions.

At the 7-day follow-up visit, questionnaires were collected, and the surgeon performed a careful examination; any existing complications or signs of impaired healing in the treated area were reported in the patient’s record.

### Data collection

Demographic information, including age, gender, smoking habits, systemic health, and occupation were collected from the CRFs. The patients’ systemic health conditions were classified according to the American Society of Anesthesiologists Physical status (ASA-PS).

Information relating to the surgical procedure included the following: the type of surgery, arch involved, site involved, the extension of the surgical site (number of teeth involved), buccal and or palatal/lingual flap involvement, vertical releasing incisions and location (mesial and/or distal), and execution of a distal wedge. In case of periodontal plastic surgery with a connective tissue graft, the palatal harvesting technique, dimension of the graft, and use of periodontal dressing were recorded. In case of regenerative procedures, information regarding biomaterials (enamel matrix derivative, bone substitute, membrane) was collected. Additional information gathered included surgeon experience, duration of the surgery, number of surgical assistants, and whether intraoral pictures were performed.

### Sample size calculation

The sample size, namely 154 subjects, was set by fixing a test power of no less than 90% associated with a significance of no more than 5%. This sample size calculation was performed using the effect size estimation from a previously published research study regarding scales of quality of life and pain [18, 29, 31, 32].

### Statistical analysis

Demographic and clinical parameters and scales have been summarized using classic descriptive statistics. A multiple linear regression with stepwise selection was fitted for the entire study population to evaluate the relationship of OHIP scale (analyzed as total OHIP-14 score and score of 7 OHIP-14 subdomains) and VAS scale and the following variables: sex (male/female), age (<50/>50), smoking (no/yes), employment (public employee/private employee/retired/student/unemployed), surgeon experience (expert/resident), duration of surgery (<60 min/>60 min and <120 min/>120 min), number of surgical assistants (one/two/three or more), execution of intraoral pictures (no/yes), arch location (maxilla/mandible), site location (posterior/anterior/both), number of teeth involved (singular/multiple), flap involvement (vestibular/palatal/both), flap extension (<3 teeth/>3 teeth), presence of releasing incisions (yes/no), palatal graft harvest (yes/no) and site (premolar/molar/mixed/tuber), and presence of periodontal dressing (yes/no). A linear model was fitted to evaluate any relationship between analgesic consumption and OHIP and VAS records.

Post-surgical complications (bleeding, swelling, eating discomfort, speech discomfort) were analyzed as dichotomous (yes/no), numeric (duration in days), and ordinal (on the basis of the likert scale) variables. Non-parametric tests (Chi-square analysis and Kruskall-Wallis test) were used to evaluate any significant between-group differences. A multiple logistic regression with stepwise selection was used to evaluate the relationship between post-surgical complications and the previously described variables. *p* values <.05 were considered to reflect a statistical significance for all analyses.

## Results

### Characteristics of the study subjects

The study population included 155 subjects who received periodontal surgery (41 males, 114 females; mean age 45.03±13.7, range 18–81). The surgeries were classified into three categories: 40 patients received resective periodontal surgery, 28 subjects received regenerative periodontal surgery, and 87 patients mucogingival surgery. Table 1 summarizes the sociodemographic, clinical, surgical characteristics, and post-surgical drug therapies of the three different treatment modalities.

**Table 1:** Sociodemographic and clinical characteristics of the study population according to the different surgical treatments.

	Resective periodontal treatment	Regenerative periodontal treatment	Mucogingival treatment	Total
No. of patients	40	28	87	155
Age	42.7±12.9	52.6±12.8	43.7±13.6	45.03±13.7
Gender	29 females 11 males	15 females 13 males	70 females 17 males	114 females 41males
Smoke	6 yes 34 no	7 yes 21 no	10 yes 77 no	23 yes 132 no
Employment	22 public employee 11 private employee 3 retired 4 student 0 unemployed	17 public employee 5 private employee 6 retired 0 student 0 unemployed	52 public employee 17 private employee 8 retired 9 student 1 unemployed	91 public employee 33 private employee 17 retired 13 student 1 unemployed
ASA status	31 ASA 1 9 ASA 2	21 ASA 1 7 ASA 2	74 ASA 1 13 ASA 2	126 ASA 1 29 ASA 2
Surgeon experience	30 expert 10 resident	25 expert 3 resident	82 expert 5 resident	137 expert 18 resident
Duration of surgery	18 <60 min 18 >60 min <120 min 4 >120 min	10 <60 min 15 >60 min <120 min 3 >120 min	39 <60 min 36 >60 min <120 min 12 >120 min	67 <60 min 69 >60 min <120 min 19 >120 min
No. of surgical assistants	17 one assistant 13 two assistants 10 three or more	11 one assistant 7 two assistants 10 three or more	20 one assistant 44 two assistants 23 three or more	48 one assistant 64 two assistants 43 three or more
Intra-oral pictures	20 yes 20 no	15 yes 13 no	78 yes 9 no	113 yes 42 no
Arch location	28 maxilla 12 mandible	8 maxilla 20 mandible	41 maxilla 46 mandible	77 maxilla 78 mandible
Site location	4 anterior 33 posterior 3 both	7 anterior 17 posterior 4 both	32 anterior 28 posterior 27 both	43 anterior 78 posterior 34 both
N° of teeth involved	9 Single 31 multiple	14 Single 14 multiple	31 Single 56 multiple	54 Single 101 multiple
Flap extension	20 <3 teeth 20 >3 teeth	13 <3 teeth 15 >3 teeth	61 <3 teeth 26 >3 teeth	94 <3 teeth 61 >3 teeth
Releasing incisions	21 yes 19 no	4 yes 24 no	67 yes 20 no	92 yes 63 no
Palatal graft	0 yes 40 no	3 yes 25 no	81 yes 6 no	84 yes 71 no
Graft site		2 molar 1 mixed	5 premolar 43 molar 31 mixed 2 tuber	5 premolar 45 molar 32 mixed 2 tuber
Periodontal dressing	0 yes 40 no	1 yes 27 no	61 yes 26 no	62 yes 93 no
Antibiotic therapy	20 yes 20 no	1 yes 27 no	62 yes 25 no	83 yes 72 no
NSAID therapy	36 anti-inf 4 anti-inf+cort	28 anti-inf	79 anti-inf 8 anti-inf+cort	143 anti-inf 12 anti-inf+cort
NSAID dosage (for 7 days)	4.97±2.5 tablets × 7 days	3.96±0.7 tablets × 7 days	4.63±2.1 tablets × 7 days	4.6±2.03 tablets × 7 days

### Perception of the postoperative course of periodontal surgery: OHIP-14 scale and VAS scale evaluation

A mean OHIP-14 total score of 9.87±8.5 (range 0–42) was calculated in the study population of 155 patients who underwent periodontal surgery. Multilevel mixed logistic regression analysis revealed a significant influence of female sex (*p *< 0.05), flap extension (*p *< 0.05), and periodontal dressing (*p *< .0.05) in the OHIP-14 scores. No significant differences were found among the three surgical groups (Tables 2 and 3).

**Table 2:** Multilevel mixed logistic regression exploring factors associated with OHIP-14 score

Groups		No. of cases (%)	OHI-P	Coefficient	95%CI	*p* value
Demographic characteristics						
Sex	Male	41 (26.5%)	6.02 ± 4.8	0.854	(0.175; 1.533)	0.014
	Female	114 (73.5%)	11.3 ± 9.1			
Age	<50	102 (65.8%)	9.84 ± 8.5	−0.010	(−0.033; 0.013)	0.389
	>50	53 (34.2%)	9.9 ± 8.4			
Employment	Public employee	91 (58.7%)	9.62 ± 7.9	0.014	(−0.271; 0.299)	0.922
	Private employee	33 (21.3%)	10.24 ± 10.5			
	Retired	17 (10.9%)	9.12 ± 7.1			
	Student	13 (8.4%)	12 ± 9.2			
	Unemployed	1 (0.6%)	6			
Smoke	No	132 (85.2%)	9.89 ± 8.5	0.163	(−0.779; 1.105)	0.734
	Yes	23 (14.8%)	9.74 ± 8.7			
Clinical and surgical characteristics						
Periodontal surgical treatment	Resective	40 (25.8%)	8.8 ± 8.3	1.765	(−1.357; 4.887)	0.268
	Regenerative	28 (18.1%)	7.8 ± 7.1			
	Muco-gingival	87 (56.1%)	11.1 ± 8.9			
Surgeon experience	Expert	137 (88.4%)	8.31 ± 8.3	0.669	(−0.456; 1.795)	0.243
	Resident	18 (11.6%)	9.72 ± 9.7			
Duration of surgery	<60	67 (43.2%)	9.82 ± 7.9	0.057	(−0.424; 0.538)	0.816
	>60 <120	69 (44.5%)	8.72 ± 8.2			
	>120	19 (12.3%)	14.21 ± 10.5			
No. of surgical assistants	One	48 (31%)	7.46 ± 7.2	0.2484	(−0.196; 0.693)	0.274
	Two	64 (41.3%)	11.88 ± 9.5			
	Three or more	43 (27.7%)	9.58 ± 7.7			
Intra-oral pictures	No	42 (27.1%)	8.02 ± 8.4	0.258	(−0.609; 1.126)	0.560
	Yes	113 (72.9%)	10.56 ± 8.5			
Arch location	Maxilla	77 (49.7%)	9.9 ± 8.4	−0.141	(−0.749; 0.466)	0.647
	Mandible	78 (50.3%)	9.8 ± 8.6			
Site location	Anterior	43 (27.7%)	13.02 ± 9.4	−0.265	(−0.705; 0.175)	0.238
	Posterior	78 (50.3%)	7.13 ± 7.3			
	Both	34 (22%)	12.18 ± 8.1			
No. of teeth involved	Singular	54 (34.8%)	8.02 ± 8.4	0.684	(−0.03; 1.40)	0.061
	Multiple	101 (65.2%)	10.89 ± 8.4			
Palatal flap	No	93 (60%)	10.58 ± 8.6	0.788	(−1.283; 2.858)	0.456
	Yes	62 (40%)	8.81 ± 8.3			
Flap extension	<3 teeth	94 (60.6%)	8.6 ± 8.12	0.954	(0.25; 1.659)	0.008
	> 3 teeth	61 (39.4%)	11.8 ± 8.78			
Releasing incisions	No	63 (40.6%)	9.2 ± 8.2	0.653	(−0.07; 1.378)	0.078
	Yes	92 (59.4%)	10.4 ± 8.7			
Palatal graft	No	71 (45.8%)	8.54 ± 7.8	0.822	(−1.513; 3.157)	0.490
	Yes	84 (54.2%)	11 ± 8.9			
Periodontal dressing	No	93 (60%)	8.66 ± 7.8	0.812	(−0.133; 1.757)	0.093
	Yes	62 (40%)	11.69 ± 9.3			
Drug consumption						
Antibiotic therapy	No	46 (29.7%)	8.37 ± 6.4	0.136	(−0.544; 0.816)	0.695
	Yes	109 (70.3%)	10.5 ± 9.2			
Anti-inflammatory drug consumption	NSAID	140 (90.3%)	10.2 ± 8.7	0.733	(−0.415; 1.88)	0.211
	NSAID + corticosteroid therapy	12 (9.7%)	6.8 ± 5.7

**Table 3:** Multiple linear logistic regression after stepwise selection for factors associated with higher OHIP-14 score

Groups	Coefficient	95%CI	*p* value
Sex	0.832	(0.210; 1.453)	0.009
Flap extension	0.917	(0.313; 1.522)	0.003
Periodontal dressing	0.872	(0.281; 1.463)	0.004

Mean values of 7 sub-domains of the OHIP-14 scale have been calculated (Supplementary information Table S1). Multilevel mixed logistic regression analysis of scores of 7 sub-domains of OHIP-14 scale revealed that female sex was a variable significantly related to functional limitation (significantly higher scores obtained from Q1 and Q2 of OHIP-14 scale), psychological discomfort (Q5 and Q6), physical disability (Q7 and Q8), psychological disability (Q9 and Q10), and social disability (Q11 and Q12). Flap extension of more than three teeth was significantly related to physical pain (Q3 and Q4), physical disability (Q7 and Q8), psychological disability (Q9 and Q10), and handicap (Q13 and Q14). The presence of a periodontal dressing was significantly related to the functional limitation subdomain (Q1 and Q2), psychological disability (Q9 and Q10), and social disability. Finally, multilevel mixed logistic regression analysis showed a significant relationship between social disability (Q11 and Q12) and the execution of a palatal graft.

The postoperative intensity of pain has been evaluated using VAS scale. A mean VAS score of 2.96±2.39 (range 0–9) was calculated in the study population of 155 patients who underwent periodontal surgery. Multilevel mixed logistic regression analysis revealed the influence of vertical releasing incisions (*p *< 0.05) and palatal flap extension (*p *< 0.05) in the VAS score. No significant differences among the three different groups of periodontal surgery were found (*p *= ns) (see Supplementary information Table S2-S3).

**Fig. 1:**
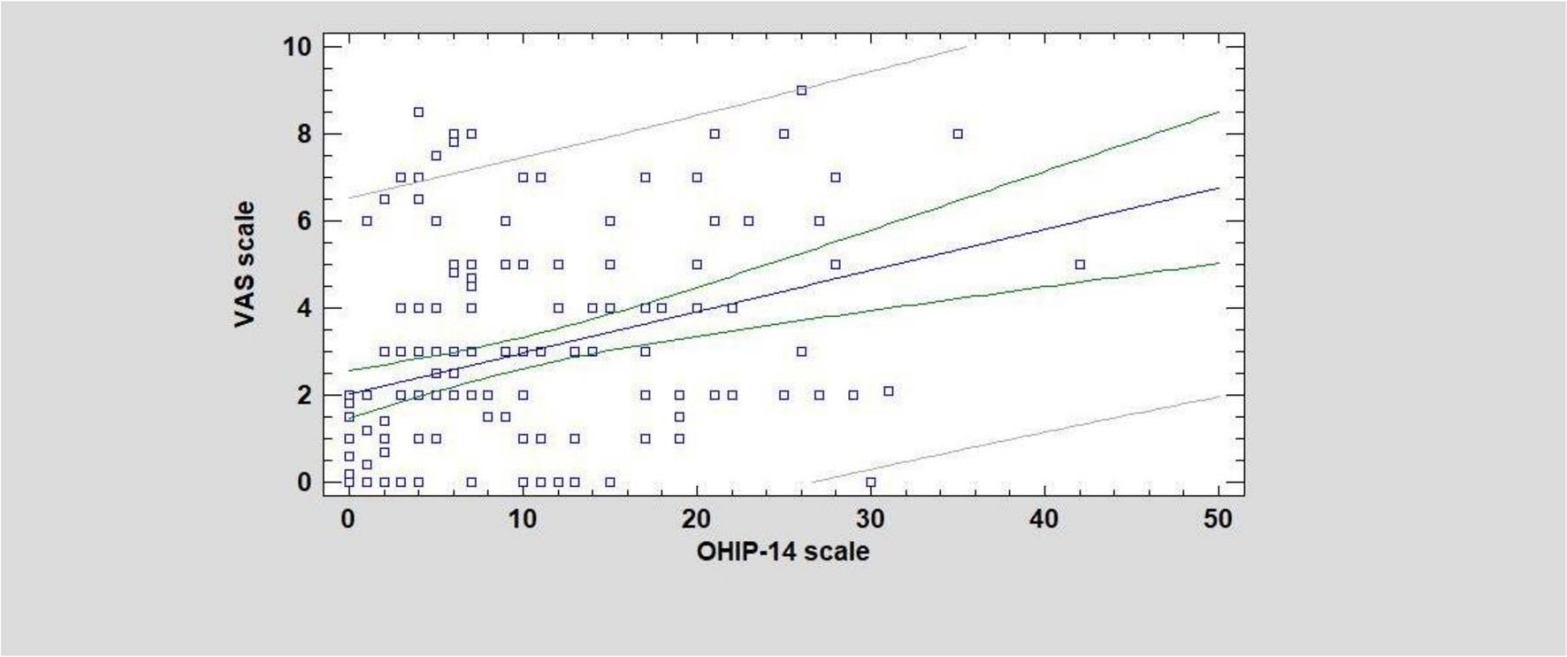
Relationship between OHIP-14 scale and VAS scale

The linear regression model showed a significant relationship between higher values of OHIP-14 and VAS scores (*F*:19.52; *r*:0.336; *p *< 0.05) (Figure 1).

Linear regression model did not show a significant relationship between post-surgical analgesic dosage (expressed as the cumulative number of NSAID tablets assumed after periodontal surgery) and OHIP-14 (*F*: 3.88; *r*:0.159; *p *= ns) and VAS records (*F*:0.05; *r*:0.02; *p *= ns).

### Post-surgical complications

Of the 155 subjects who received periodontal surgery, 40 (25.8%) patients reported bleeding as post-surgical complication, 96 (61.9%) reported swelling, 105 (67.7%) reported eating discomfort, and 44 (28.4%) patients reported speech discomfort.

Table 4 describes the distribution of post-surgical complications in the three different periodontal surgical treatments and the severity of post-surgical complications on the basis of 5-point Likert scale questionnaire. Furthermore, Table 5 reports the duration in days of post-surgical complications.

**Table 4:** Between-groups distribution of post-surgical complications on the basis 5-point Likert scale for bleeding, swelling, eating discomfort, and speech discomfort

	Resective periodontal surgical treatment	Regenerative periodontal treatment	Muco-gingival periodontal treatment	Test (*p* value)
Bleeding				
No. 40/155 cases	11/40 cases	4/28 cases	25/87 cases	*p*=.36
Far too little	1	0	0	*p*=.8
Too little	6	2	12	
About right	4	2	11	
Too much	0	0	1	
Far too much	0	0	1	
Swelling				
No. 96/155 cases	23/40 cases	14/28 cases	59/87 cases	*p=.*14
Far too little	8	7	16	*p=.*7
Too little	9	5	29	
About right	5	2	12	
Too much	0	0	0	
Far too much	1	0	2	
Eating discomfort				
No. 105/155 cases	25/40 cases	20/28 cases	60/87 cases	*p= .*69
Far too little	2	3	6	*p=*.5
Too little	7	10	18	
About right	12	5	21	
Too much	2	2	7	
Far too much	2	0	8	
Speech discomfort				
44/155 cases	8/40 cases	8/28 cases	28/87 cases	*p=.*36
Far too little	3	0	6	*p=.*6
Too little	1	4	11	
About right	3	2	7	
Too much	0	0	1	
Far too much	1	2	3

**Table 5:** Duration in days for bleeding, swelling, eating discomfort, and speech discomfort

	Resective periodontal surgical treatment	Regenerative periodontal treatment	Muco-gingival periodontal treatment	Test (*p* value)
Bleeding	2.1±1.7	1.05 ± 0.7	1.8 ± 1.4	*p=*0.4
Swelling	3.3±1.7	2.8 ± 1.6	3.5 ± 1.9	*p=*0.4
Eating discomfort	4.8±2.2	4.6 ± 1.8	4.9 ± 2.1	*p=*0.7
Speech discomfort	3.8±2.5	3.9 ± 2.1	4 ± 3.1	*p=*0.9

The three different periodontal surgical groups did not significantly differ in terms of distribution, severity, and duration of post-surgical complications (*p *= ns, see details in Tables 4 and 5).

### Risk factors associated with post-surgical complications

Multiple logistic regression showed that none of the analyzed variables is significantly related to post-surgical presence of bleeding.

Duration of surgery (Chi 6.9; *p *< 0.05) and sex (Chi 9.5; *p *< 0.05) are the only variables significantly related to the presence of swelling after periodontal surgery.

Flap extension (Chi 5.3; *p *< .05) was the only variable significantly related to eating discomfort after periodontal surgery.

Sex (Chi 18.6; *p *< .05), flap extension (Chi 5.1; *p *< .05), vertical releasing (Chi 7.6; *p *< .05), and number of teeth involved (Chi 7.9; *p *< .05) were variables significantly related to speech discomfort.

## Discussion

The present observational cohort study was performed to investigate the impact of different periodontal surgical treatments on patient quality of life by means of the OHIP-14 questionnaire.

Other studies have shown that patients’ pain scores recorded on the day of surgery were significantly correlated with those reported at 24 h and at 1-week post-surgery [33]. Therefore, it is reasonable to interpret the results at 7 days as an overall impression of pain and discomfort that the patients perceived in the former days, mostly representing the peak intensity that the patient could recall. For this reason, in this study, we decided to take into account the information regarding the 1-week postoperative time point.

Our findings showed that the OHIP-14 score was very low 9.87±8.5 (range 0–42) in all patients who underwent periodontal surgery; thus, indicating that periodontal surgical treatment is well tolerated by patients and has a low influence on the quality of life.

When the OHIP-14 scores were analyzed, physical pain, physical disability, and functional limitation were the most impaired subdomains at 7 days after surgery. Furthermore, three factors were found to be significantly correlated with higher OHIP-14 scores: flap extension, the application of the periodontal dressing, and gender (female sex).

Flap extension of more than three teeth reported a mean score value of 11.8±8.78, impacting the overall postoperative pain. This finding is confirmed by previous studies [10, 34–37] in which larger surgical areas resulted in higher postoperative morbidity. It is possible to assume that wider areas of bone and periosteum exposed during surgery could negatively impact the blood supply and account for increased edema and bleeding, as well as altered healing [7].

Use of a periodontal dressing was associated with significantly higher values of OHIP after stepwise logistic regression (*p*<.05), impacting significantly the functional limitation subdomain (Q1 and Q2), psychological disability (Q9 and Q10), and social disability (Q11 and Q12). Presumably, the volume of the periodontal dressing, intended for the protection of palatal wounds, may create more discomfort for patients during routine chewing and speech. Therefore, the higher OHIP-14 score could be related to the dressing’s dimension. As a matter of fact, no correlation was found regarding the application of periodontal dressing influencing the VAS pain score, which is in line with our OHIP-14 results. Likewise, several studies in the literature have reported that using periodontal dressing on the palatal donor site reduces pain perceived by patients, reporting a psychological feeling of protection and well-being with its use [38–41]; however, there was no mention regarding the “bulk” of the periodontal dressing creating discomfort. Nowadays, periodontal dressing is being widely replaced by the use of flatter protection represented by cyanoacrylate (alone or associated with a collagen sponge and sutures), which, apart from being well tolerated by patients, has been reported to result in effective hemostasis, analgesia, and wound healing promotion [40].

In the present study, the female gender has been linked with higher scores of OHIP-14 in terms of functional limitation (Q1 and Q2), psychological discomfort (Q5 and Q6), physical disability (Q7 and Q8), psychological disability (Q9 and Q10), and social disability (Q11 and Q12). These findings are in contrast with data present in the literature [10, 29], in which no statistically significant difference was reported in terms of postoperative pain perception. It may be speculated that this difference is due to the cohort nature of this study, in which females and males are not homogeneously represented.

The overall incidence of post-surgical pain and infection is low following periodontal and implant surgery, and the intensity is mild for the majority of patients [5, 10, 12, 35]. In general, this study showed better or comparable pain perception findings than previous studies on periodontal surgery [3, 7, 8, 11, 12, 18, 29]. A study by Ozcelik et al. [18] reported OHIP values of 27.5 for open flap surgery and a score of 12 for regenerative surgery, at 7 days postoperatively. In the present study, the highest OHIP score was for mucogingival surgery (11.06±8.9), followed by resective (8.8±8.3) and regenerative surgery (7.8±7.1) in the last place. A study by Tonetti et al. [29] evaluating PROMS after mucogingival surgery performed by experienced clinicians reported OHIP scores of 9.3±9.7 at 7 days. In the current study, the mean OHIP score for all surgeries performed only by expert clinicians was comparable (8.31±8.3).

While similar results have been observed in another study using both OHIP-14 and VAS pain scores to evaluate postoperative PROMs after periodontal surgery [29], to our knowledge, this is the first study to confirm the presence of a significant relationship between higher values of OHIP-14 and higher VAS scores.

Based on our findings, there appears to be a trend of lower PROMs regarding pain and postoperative discomfort after periodontal surgery. This could shed light on the evolution of the surgical techniques themselves, which have become less invasive throughout the years surely aided by the standardized use of magnification, and refinement of instruments, sutures, and dressing materials. In this regard, it is worth noting that in our study, there was no statistically significant difference between OHIP-14 and VAS scores of the three surgical modalities. This may also be a result of the aforementioned factors leading to a very similar postoperative course, even for mucogingival surgery which was once regarded as one of the most invasive periodontal surgical procedures.

Post-surgical complications represent a matter of concern for both patients and clinicians, even if this is not always synonymous with bad wound healing. In the literature, the most reported complications after periodontal surgery are swelling, bleeding, bruising, eating, and speech discomfort. Swelling and bruising are normal post-surgical events that may occur the days after surgery, while bleeding is not a common complication, and it can happen especially after harvesting procedures from the palate [42, 43].

The known factors associated with postoperative discomfort, swelling, or bleeding, based on previous evidence, include but are not limited to, patient age [11, 44], patient gender [14, 34, 45], smoking [46], previous experience of surgery [36], treatment by an experienced periodontist [13], the duration of the surgical procedure [7, 9], the involvement of vertical releasing incisions or periosteal fenestration [47], the use of periodontal dressing [39, 48], and the presence of diabetes [49].

Even though more than half of the patients in this study reported postoperative complications (Table 4), it should be noted that their intensity was very low, and the duration was limited to a few days (Table 5). This is in line with the present OHIP-14 score confirming the limited impact of periodontal surgery on patients’ quality of life. Furthermore, there was no statistical difference among the three surgical modalities in terms of incidence, severity, and duration of post-surgical complications. None of the analyzed demographic or clinical variables was significantly related to post-surgical bleeding occurrence; however, bleeding was more frequent after mucogingival surgery.

Regarding post-surgical discomfort, flap extension was the only variable related to eating limitations; while gender, number of teeth involved, and flap extension were the most determining factors influencing speech impairment.

## Conclusion

Given the nature of the protocol (cohort observational study), one of the major limitations in this study is the unbalanced distribution of the evaluated parameters (sex, type of surgery, and surgeon’s experience). Nevertheless, based on our findings, it is possible to extrapolate that periodontal surgical procedures have a low impact on patients’ quality of life as evaluated through the OHIP-14 and VAS pain questionnaires. More so, the incidence of post-surgical complications is not significant, and the duration of postoperative discomfort is very limited (less than 7 days). Further investigations with controlled groups are strongly advocated.

### Supplementary Information

Below is the link to the electronic supplementary material.

Supplementary file1 (PDF 138 kb)

## Data Availability

All data supporting this study are included within the article.
