# Supplementary material for: Patient-reported outcome measures after periodontal surgery
Source: Clin Oral Investig. 2023 Nov 9;27(12):7715–24. doi: 10.1007/s00784-023-05362-y (PMC10713745; doi:10.1007/s00784-023-05362-y)
Supplement: Supplementary file 1 — (PDF 138 kb) [file 784_2023_5362_MOESM1_ESM.pdf]

Sub-domains of OHIP-14 scale	Resective periodontal surgery	Regenerative periodontal surgery	Muco-gengival periodontal surgery	TOTAL
1-Functional limitation (Q1-Q2)	1.3±1.7	0.75±1.3	1.41±1.6	1.26±1.6
2-Physical pain (Q3-Q4)	2.58±1.9	2.46±1.8	2.6±2.1	2.58±1.9
3-Psychological discomfort (Q5-Q6)	0.95±1.5	0.64±1.2	1.44±1.8	1.17±1.7
4-Physical disability (Q7-Q8)	1.48±1.76	1.14±1.4	1.79±1.8	1.59±1.7
5-Psychological disability (Q9-Q10)	0.77±1.31	0.61±1.03	1.15±1.34	0.95±1.3
6-Social disability (Q11-Q12)	0.85±1.2	1.07±1.5	1.26±1.6	1.12±1.5
7-Handicap (Q13-14)	0.83±1.3	1.1±1.5	1.4±1.7	1.2±1.6
OHIP-14 total score	8.75±8.3	7.78±7.1	11.06±8.9	9.87±8.5

Table S1: OHIP-14 scale and subdomains mean values

**Groups**		**N of cases (%)**	**VAS**	**Coefficient**	**95%CI**	**P value**
**Demographic characteristics**						
Sex	Male	41 (26.5%)	2.99±2.5	0.329	(-0.399; 1.058)	0.376
	Female	114 (73.5%)	2.87±2.2			
Age	<50	102 (65.8%)	2.85±2.6	-0.009	(-0.033; 0.013)	0.399
	>50	53 (34.2%)	2.99±2.3			
Employment	Public employee	91 (58.7%)	2.94±2.4	0.15	(-0.154; 0.454)	0.335
	Private employee	33 (21.3%)	3.04±2.5			
	Retired	17 (10.9%)	3.05±2.5			
	student	13 (8.4%)	2.45±2.2			
	unemployed	1 (0.6%)	3.16±1.9			
Smoke	No	132 (85.2%)	3.17±2.6	0.043	(-0.834; 0.92)	0.924
	Yes	23 (14.8%)	5			
**Clinical and surgical characteristics**						
Periodontal surgical treatment	Resective	40 (25.8%)	3.7±2.9	-0.057	(-0.820; 0.706)	0.883
	Regenerative	28 (18.1%)	2.9±2.4			

	Muco-gengival	87 (56.1%)	2.6±2		[illegible]	
Surgeon experience	Expert	137 (88.4%)	2.83±2.3	0.643	(-0.449; 1.734)	0.249
	resident	18 (11.6%)	3.94±2.8			
Duration of surgery	<60	67 (43.2%)	2.67±2.2	0.233	(-0.25; 0.715)	0.345
	>60 <120	69 (44.5%)	3.01±2.4			
	>120	19 (12.3%)	3.76±2.8			
N° of surgical assistants	One	48 (31%)	3.08±2.7	0.15	(-0.299; 0.599)	0.513
	Two	64 (41.3%)	3.02±2.2			
	Three or more	43 (27.7%)	2.72±2.4			
Intra-oral pictures	No	42 (27.1%)	3.35±2.7	-0.214	(-1.127; 0.698)	0.645
	yes	113 (72.9%)	2.81±2.3			
Arch location	Maxilla	77 (49.7%)	2.93±2.6	0.235	(-0.361; 0.831)	0.439
	mandible	78 (50.3%)	2.98±2.2			
Site location	Anterior	43 (27.7%)	2.71±1.9	-0.48	(-1.069; 0.109)	0.110
	Posterior	78 (50.3%)	3.05±2.7			
	both	34 (22%)	3.06±2.3			
N° of teeth involved	Singular	54 (34.8%)	2.55±2.2	0.434	(-0.271; 1.14)	0.228
	multiple	101 (65.2%)	3.17±2.5			
Palatine flap	No	93 (60%)	2.55±1.9	1.467	(-0.285; 3.219	0.101
	Yes	62 (40%)	3.57±2.8			
Flap extension	<3 teeth	94 (60.6%)	2.85±2.4	0.27	(-0.421; 0.961)	0.444
	> 3 teeth	61(39.4%)	3.12±2.3			
Vertical releasing	No	63 (40.6%)	2.83±2.5	0.812	(0.0833; 1.541)	0.029
	yes	92 (59.4%)	3.03±2.3			
Palatine graft	No	71 (45.8%)	3.3±2.7	1.467	(-0.285; 3.219)	0.101
	Yes	84 (54.2%)	2.67±2.1			
Periodontal dressing	No	93 (60%)	3.18±2.6	0.088	(-0.835; 1.011)	0.852
	yes	62 (40%)	2.62±1.9			
**Drug consumption**						
Antibiotic therapy	No	46 (29.7%)	3.43±2.5	-0.284	(-0.966; 0.398)	0.415
	yes	109 (70.3%)	2.76±2.3			
NSAID therapy	NSAID	140 (90.3%)	3.04±2.4			

	NSAID + corticosteroid therapy	12 (9.7%)	2.2±1.8	-0.673	(-1.883; 0.537)	0.276

Table S2: multilevel mixed logistic regression exploring factors associated with VAS score

Groups	Coefficient	95%CI	P value
Presence of Palatine flap	0.802	(0.1672; 1.437)	0.013
Vertical releasing	1.074	(-0.068; 2.092)	0.04

Table S3: multiple linear logistic regression after stepwise selection for factors associated with higher VAS score
